# Molecular interplay between leptin, insulin-like growth factor-1, and β-amyloid in organotypic slices from rabbit hippocampus

**DOI:** 10.1186/1750-1326-6-41

**Published:** 2011-06-08

**Authors:** Gurdeep Marwarha, Jaya RP Prasanthi, Jared Schommer, Bhanu Dasari, Othman Ghribi

**Affiliations:** 1 Department of Pharmacology, Physiology and Therapeutics, University of North Dakota School of Medicine and Health Sciences, Grand Forks, North Dakota, 58202, USA

**Keywords:** Leptin, IGF-1, Aβ42, mTORC1, C-EBPα, STAT5, Organotypic slices

## Abstract

**Background:**

Evidence shows that the insulin-like growth factor-1 (IGF-1) and leptin reduce β-amyloid (Aβ) production and tau phosphorylation, two major hallmarks of Alzheimer's disease (AD). IGF-1 expression involves the JAK/STAT pathway and the expression of leptin is regulated by the mammalian target of rapamycin complex 1 (mTORC1). We have previously shown that Aβ reduces leptin by inhibiting the mTORC1 pathway and Aβ was also suggested to inhibit the JAK/STAT pathway, potentially attenuating IGF-1 expression. As IGF-1 can activate mTORC1 and leptin can modulate JAK/STAT pathway, we determined the extent to which IGF-1 and leptin can upregulate the expression of one another and protect against Aβ-induced downregulation.

**Results:**

We demonstrate that incubation of organotypic slices from adult rabbit hippocampus with Aβ42 downregulates IGF-1 expression by inhibiting JAK2/STAT5 pathway. Leptin treatment reverses these Aβ42 effects on IGF-1 and treatment with the STAT5 inhibitor completely abrogated the leptin-induced increase in IGF-1. Furthermore, EMSA and ChIP analyses revealed that leptin increases the STAT5 binding to the IGF-1 promoter. We also show that IGF-1 increases the expression of leptin and reverses the Aβ42-induced attenuation in leptin expression via the activation of mTORC1 signaling as the mTORC1 inhibitor rapamycin completely precluded the IGF-1-induced increase in leptin expression.

**Conclusion:**

Our results demonstrate for the first time that Aβ42 downregulates IGF-1 expression and that leptin and IGF-1 rescue one another from downregulation by Aβ42. Our study provides a valuable insight into the leptin/IGF-1/Aβ interplay that may be relevant to the pathophysiology of AD.

## Background

Alzheimer's disease (AD) is pathologically characterized by the deposition and accumulation of β-amyloid (Aβ) peptide in extracellular plaques, the deposition of hyperphosphorylated tau in intracellular neurofibrillary tangles (NFT's), oxidative stress and synaptic loss. Increased levels of Aβ42 (soluble and insoluble) are suggested to play a key role in the neurodegenerative processes that characterize AD. Reduction in the accumulation of this peptide is widely viewed as a potential strategy to protect against AD. There is compelling evidence that the insulin-like growth factor-1 (IGF-1) is involved in the metabolism and clearance of Aβ [1,2]. Several studies have shown that serum levels of IGF-1 are decreased in AD patients [3-5]. IGF-1 is endogenously produced in the central nervous system [6-8] and is also transported into the brain from the periphery across the blood-brain barrier [9]. In the peripheral system, IGF-1 expression is contingent on the activation of the JAK/STAT pathway, involving the transcription factor STAT5 [10,11].

Leptin, an adipocytokine produced endogenously in the brain [12-15], has also been shown to reduce Aβ levels *in vitro *[16] as well as *in vivo *[17,18] and circulating leptin levels are reduced in AD [19]. Expression levels of leptin are regulated by the mammalian target of rapamycin complex 1 (mTORC1) [20-22]. Interestingly, IGF-1 and leptin are interconnected. While IGF-1 activates mTORC1 [23,24], potentially increasing expression levels of leptin, numerous studies have demonstrated the activation of STAT5 by leptin [25-28] suggesting that leptin may control IGF-1 expression via STAT5 activation. We have recently demonstrated that Aβ42 downregulates leptin expression levels in organotypic hippocampal slices via inhibition of the mTORC1 signaling pathway [15]. However, the extent to which Aβ42 may inhibit IGF-1 expression by inhibiting JAK2/STAT5 has not been determined. Furthermore, the extent to which IGF-1 treatment activates mTORC1 and treatment with leptin activates JAK2/STAT5 respectively precluding Aβ42-induced leptin and IGF-1 downregulation are not known. In this study we found that Aβ42 reduces IGF-1 expression levels by inhibiting JAK2/STAT5 pathway and treatment with leptin prevented these Aβ42 effects. IGF-1 treatment also upregulated leptin levels and prevented Aβ42-induced leptin downregulation by mechanisms involving mTORC1 activation. As increased levels of Aβ42 is a major pathogenic factor in AD, understanding the cellular mechanisms by which IGF-1 and leptin interact to modulate Aβ42 effects may be relevant to the search of agents that preclude the deleterious effects of this peptide.

## Results

### Aβ42 decreases IGF-1 expression levels and treatment with exogenous leptin reverses the effects of Aβ42

**Figure 1:**
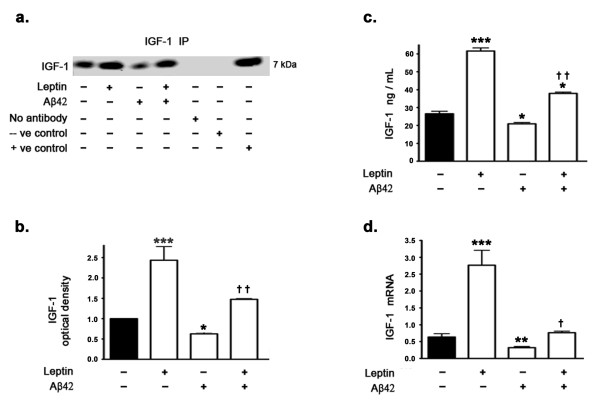
**Aβ42 reduces IGF-1 expression levels, an effect reversed by leptin treatment**. (a) Representative Western blot, (b) densitometric analyses, (c) ELISA immunoassay, and (d) Real time RT-PCR analysis show that treatment of organotypic slices with leptin for 72 hours significantly increases basal levels of IGF-1 and reverses Aβ42-induced decrease in IGF-1 expression levels. *p < 0.05, **p < 0.01 and ***p < 0.001 versus control; ^† ^p < 0.05 and ^† † ^p < 0.01 versus Aβ42.

Western blotting and densitometric analysis (Figure 1a,b) show a decrease in IGF-1 levels in the organotypic hippocampal slices treated with Aβ42 compared to untreated organotypic slices. Interestingly, treatment with leptin completely restores the decrease in IGF-1 levels induced by Aβ42. Leptin treatment also increases basal IGF-1 levels. Quantitative determination of IGF-1 levels by ELISA immunoassay (Figure 1c) corroborates Western blotting data and demonstrates that Aβ42 treatment decreases IGF-1 protein levels and concomitant treatment with leptin reverses the decrease induced by Aβ42. ELISA immunoassay also clearly depicts the increase in basal IGF-1 protein levels induced by leptin treatment. Real time RT-PCR analysis (Figure 1d) shows a significant decrease in IGF-1 mRNA in organotypic hippocampal slices treated with Aβ42 compared to untreated organotypic slices. Treatment with leptin completely restores the decrease in IGF-1 mRNA induced by Aβ42. Leptin treatment also increases the basal IGF-1 mRNA levels.

### Aβ42 attenuates JAK2/STAT5 signaling and treatment with exogenous leptin restores JAK2/STAT5 signaling

**Figure 2:**
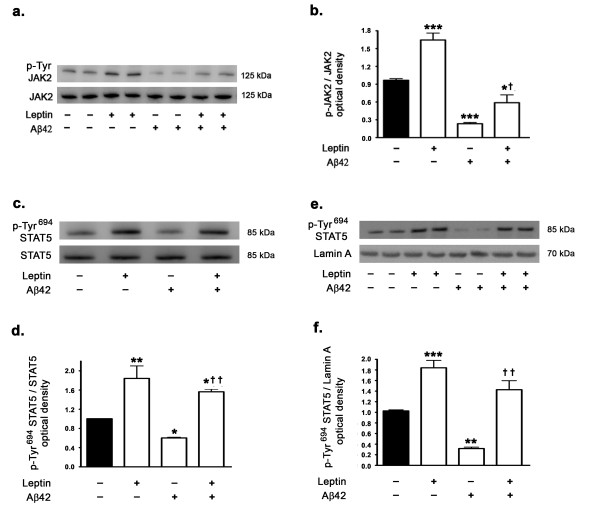
**Leptin treatment activates JAK2/STAT5 pathway and reverses the inhibition by Aβ42 of the JAK2/STAT5 activation**. (a) Representative Western blot and (b) densitometric analyses show that treatment of organotypic slices with Aβ42 for 72 hours significantly decreases phosphorylation of JAK2 at Tyr^1007/1008 ^residues. Leptin treatment increases basal levels and opposes Aβ42-induced decrease in p-Tyr^1007/1008 ^JAK2 levels. (c,d) Treatment of organotypic slices with Aβ42 for 72 hours also decreases phosphorylation of STAT5 at Tyr^694^, potentially mitigating STAT5 activation. Leptin treatment increases basal levels of p-Tyr^694 ^STAT5 and prevents the reduction in p-Tyr^694 ^STAT5 induced by Aβ42. (e,f) Treatment of organotypic slices with Aβ42 reduces the translocation of p-Tyr^694 ^STAT5 into the nucleus as evidenced by reduced levels of p-Tyr^694 ^STAT5 in the nuclear fractions. Leptin treatment, alone or in the presence of Aβ42, increases the nuclear levels of p-Tyr^694 ^STAT5. *p < 0.05, **p < 0.01 and ***p < 0.001 versus control; ^† ^p < 0.05 and ^† † ^p < 0.01 versus Aβ42.

As the JAK2/STAT5 pathway activation is involved in the regulation of peripheral IGF-1 expression and given that leptin activates the JAK2/STAT5 pathway, we determined the effects of Aβ42 on the activation status of JAK2/STAT5 in the presence and absence of leptin. Western blotting and densitometric analysis show that Aβ42 significantly attenuates JAK2/STAT5 signaling in hippocampal organotypic slices as evidenced with a decrease in p-Tyr^1007/1008 ^JAK2 (Figure 2a,b) and p-Tyr^694 ^STAT5 levels (Figure 2c,d). Leptin treatment elicited a significant increase in p-Tyr^1007/1008 ^JAK2 (Figure 2a,b) and p-Tyr^694 ^STAT5 levels (Figure 2c,d). While leptin treatment partially, yet significantly, reversed the effect of Aβ42 on p-Tyr^1007/1008 ^JAK2 it completely restored p-Tyr^694 ^STAT5 levels from the attenuation induced by Aβ42 (Figure 2c,d).

Furthermore, as the nuclear translocation and subsequent transcriptional activity of STAT5 is contingent on phosphorylation, we determined the effect of Aβ42 and leptin treatment on levels of p-Tyr^694 ^STAT5 in the nuclear extracts. We found that Aβ42 treatment completely abolished the translocation of STAT5 to the nucleus, thus mitigating STAT5 transcriptional activity (Figure 2e,f). Leptin treatment, either alone or concomitant with Aβ42, elicited a profound rise in STAT5 translocation to the nucleus (Figure 2e,f).

### Leptin induces IGF-1 expression levels via STAT5

**Figure 3:**
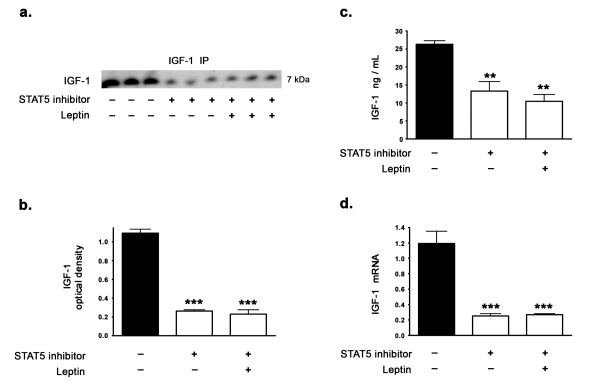
**Leptin does not regulate IGF-1 levels in the presence of a STAT5 inhibitor**. (a) Representative Western blot, (b) densitometric analysis, (c) ELISA immunoassay, and (d) Real time RT-PCR demonstrate that treatment of organotypic slices with the selective STAT5 inhibitor for 72 hours significantly decreases IGF-1 expression levels. Concomitant treatment with leptin fails to reverse the decrease in IGF-1 expression levels induced by the STAT5 inhibitor. **p < 0.01 and ***p < 0.001 versus control.

As we observed a significant increase in IGF-1 protein levels and IGF-1 mRNA expression with leptin treatment, we examined the extent to which activated STAT5 regulates IGF-1 expression levels and mediates the leptin-induced upregulation in IGF-1 expression levels in the hippocampus. To characterize the involvement of STAT5 as the mediator of leptin-induced increase in IGF-1 expression levels, we systematically treated organotypic slices with a specific inhibitor of STAT5. The STAT5 inhibitor 573108 we used has an IC_50 _of ~ 47 μM and selectively targets the SH2 domains of STAT5, preventing its phosphorylation, activation, dimerization and subsequent nuclear translocation [29]. The STAT5 inhibitor 573108 targets STAT5 specifically while eliciting no effect on STAT1 or STAT3 even at 600 μM [29]. Treatment of organotypic slices with the STAT5 inhibitor significantly attenuated IGF-1 protein levels as measured by Western blotting (Figure 3a,b) and ELISA immunoassay (Figure 3c). The STAT5 inhibitor significantly attenuated IGF-1 mRNA expression as demonstrated by real time RT-PCR (Figure 3d) suggesting the importance of STAT5 in basal and leptin-mediated increase in IGF-1 expression. Concomitant leptin treatment with STAT5 inhibitor failed to rescue the attenuated IGF-1 expression levels induced by the STAT5 inhibitor, thus suggesting that leptin induces IGF-1 expression via STAT5.

### Leptin induces IGF-1 expression levels by increasing the binding of STAT5 to the IGF-1 promoter region

**Figure 4:**
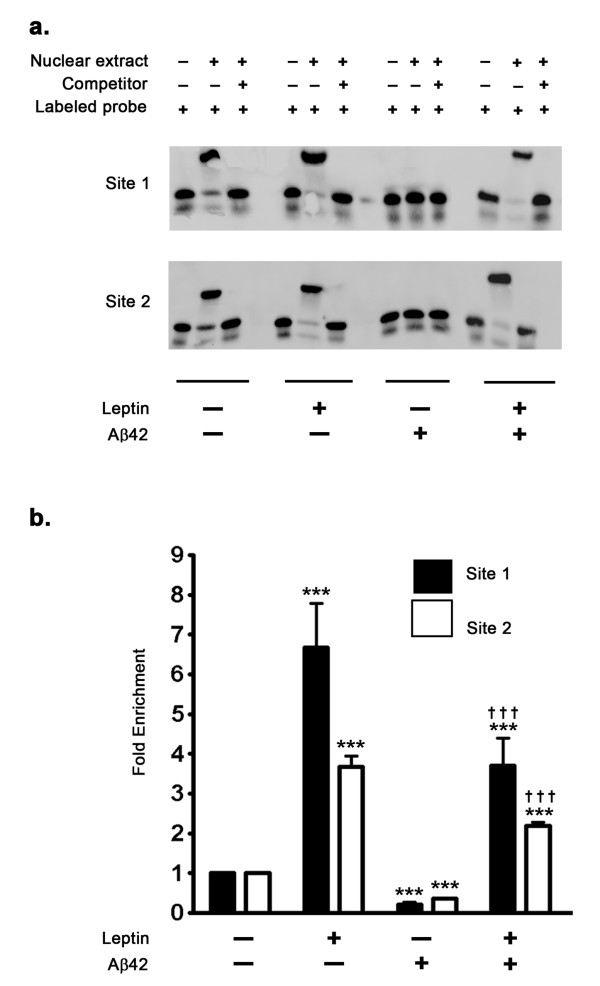
**Effects of Aβ42 and leptin on the binding of STAT5 to IGF-1 promoter region**. (a) Electrophoretic Mobility Shift Assay (EMSA) demonstrates that Aβ42 abrogates STAT5 binding to the exogenous oligonucleotide probes. Leptin treatment restores STAT5 binding to the oligonucleotide probe. (b) A Chromatin Immunoprecipitation (ChIP) assay demonstrates that treatment with leptin results in about 7-fold increase in STAT5 binding in the IGF-1 promoter region. ChIP analysis also revealed that treatment with Aβ42 attenuates binding of STAT5 to the IGF-1 promoter, while concomitant leptin treatment precludes this deleterious effect. ***p < 0.001 versus control; ^† † † ^p < 0.001 versus Aβ42.

To elucidate the mechanism of leptin-induced STAT5-mediated increase in expression levels of IGF-1 and further characterize the role of STAT5 in IGF-1 transcription, we performed an Electrophoretic Mobility Shift Assay (EMSA) with a double stranded DNA probe corresponding to the STAT5 binding consensus sequence on the rabbit IGF-1 promoter. The STAT5 binding site in the IGF-1 distal promoter region has been well characterized in humans [30] and in mouse [31]. EMSA analysis was performed using double stranded oligonucleotide probes that correspond to two evolutionary conserved STAT5 binding sites in the IGF-1 promoter region (GenBank Accession # - AF022961). EMSA (Figure 4a) analysis clearly demonstrates increased STAT5 binding to the labeled exogenous double stranded oligonucleotide probe that corresponds to the STAT5 binding site in the IGF-1 promoter region in response to leptin treatment. Furthermore, treatment with Aβ42 completely abolished STAT5 binding to this exogenous oligonucleotide probe, therefore indicating that Aβ42 attenuates STAT5 binding to the IGF-1 promoter. Co-treatment of organotypic slices with leptin and Aβ42 completely restored the STAT5 binding to the exogenous oligonucleotide probe. We next performed ChIP analysis to evaluate the extent of STAT5 binding in the IGF-1 promoter region. ChIP assay (Figure 4b) clearly shows increased STAT5 binding in the IGF-1 promoter region in response to leptin treatment as demonstrated by a 6 fold enrichment of the STAT5 binding site upon qPCR compared to control after normalization to % input. In a stark contrast, treatment with Aβ42 results in a marked loss of STAT5 binding in the IGF-1 promoter region as determined by amplification of STAT5 binding site using qPCR, thus accounting for a decrease in IGF-1 expression observed with Aβ42 treatment. Leptin treatment completely reverses the inhibitory effects of Aβ42 on STAT5 binding in the IGF-1 promoter and therefore reverses the inhibition induced by Aβ42 treatment on IGF-1 transcription.

### IGF-1 increases leptin expression levels and reverses the Aβ42-induced attenuation in leptin expression

**Figure 5:**
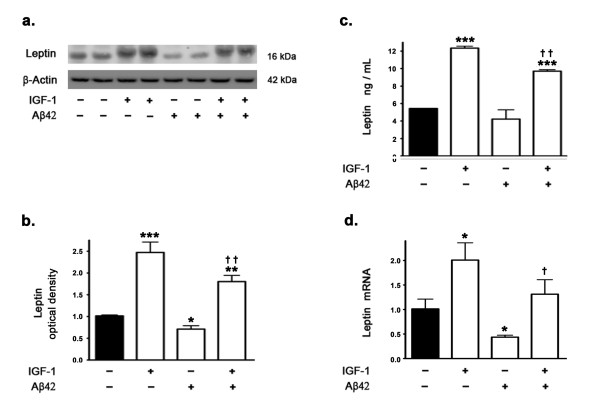
**IGF-1 reverses Aβ42-induced decrease in leptin expression levels**. (a) Representative Western blot, (b) densitometric analysis, (c) ELISA immunoassay, and (d) Real time RT-PCR show that treatment of organotypic slices with Aβ42 for 72 hours significantly decreases leptin expression levels. IGF-1 treatment increases basal leptin levels and opposes the reduction induced by Aβ42 on leptin levels. *p < 0.05, **p < 0.01 and ***p < 0.001 versus control; ^† ^p < 0.05 and ^† † ^p < 0.01 versus Aβ42.

Our previous studies demonstrated that Aβ42 decreases leptin expression levels by attenuating mTORC1 activation and signaling [15]. There is preponderance of evidence that IGF-1 activates mTORC1 signaling through IRS-1/PI3K/Akt pathway [23,24,32]. We determined the effects of IGF-1 treatment on leptin expression in the presence and absence of Aβ42. Western blotting and densitometric analysis (Figure 5a,b) show that IGF-1 treatment significantly increases the levels of leptin compared to basal levels in control untreated slices. Immunoassay using ELISA also clearly demonstrates that IGF-1 increases leptin protein levels (Figure 5c). Real time RT-PCR analysis demonstrates that IGF-1 treatment increases leptin mRNA expression (Figure 5d). Furthermore, IGF-1 treatment also completely reverses the attenuation in leptin protein levels induced by Aβ42 as demonstrated by Western blotting and densitometric analyses (Figure 5a,b) as well as by ELISA immunoassay (Figure 5c). IGF-1 treatment also completely reverses the attenuation in leptin mRNA expression induced by Aβ42 as demonstrated by real time RT-PCR analysis (Figure 5d).

### IGF-1 increases leptin expression levels via the activation of mTORC1

**Figure 6:**
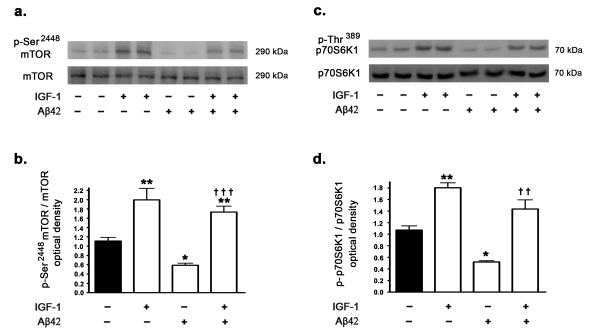
**Aβ42 inhibits mTORC1 and treatment with IGF-1 alleviates the mTORC1 inhibition**. (a) Representative Western blot and (b) densitometric analysis demonstrate that treatment of organotypic slices with Aβ42 for 72 hours significantly attenuates the phosphorylation of mTOR. IGF-1 treatment increases the phosphorylation of mTOR by 2 fold and reverses the reduction conferred by Aβ42 on mTOR phosphorylation. (c,d) Treatment of organotypic slices with Aβ42 significantly attenuates the phosphorylation of p70S6K1, the downstream substrate and indicator of mTORC1 activation. IGF-1 treatment increases phosphorylation of p70S6K1 by 1.7 fold and reverses the reduction induced by Aβ42 on p70S6K1 phosphorylation. *p < 0.05 and **p < 0.01 versus control; ^† † ^p < 0.01 and ^† † † ^p < 0.001 versus Aβ42.

**Figure 7:**
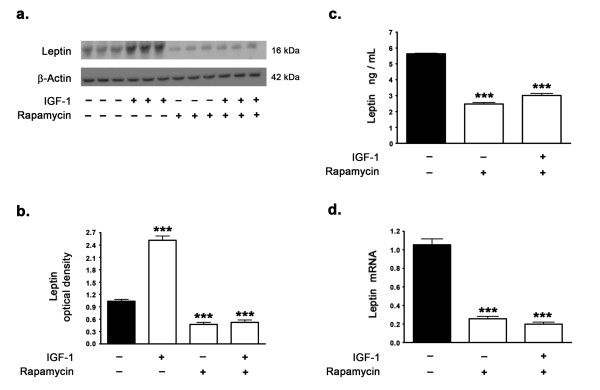
**Inhibition of mTORC1 with rapamycin prevents IGF-1-induced increase in leptin expression levels**. (a) Representative Western blot, (b) densitometric analyses, (c) ELISA immunoassay analysis, and (d) Real time RT-PCR show that treatment of organotypic slices with IGF-1 for 72 hours significantly increases basal expression levels of leptin but fails to prevent the reduction of leptin expression levels induced by rapamycin. ***p < 0.001 versus control.

As we found in this study that IGF-1 increases leptin expression levels and our previous studies have demonstrated that mTORC1 activation is a requisite for leptin expression, we determined whether IGF-1 treatment activates mTORC1 signaling. Several other studies have demonstrated that IGF-1 increases mTORC1 activation and signaling through Akt activation [33]. We determined the effects of IGF-1 on the phosphorylation status of mTOR (measuring levels of p-Ser^2448 ^mTOR) and on the phosphorylation status of p70S6K1 (p-Thr^389 ^p70S6K1), the downstream substrate and indicator of mTOR activation. Aβ42 treatment caused a significant reduction in the levels of p-Ser^2448 ^mTOR (Figure 6a,b) and p-Thr^389 ^p70S6K1 (Figure 6c,d), suggesting that treatment with Aβ42 results in downregulation of mTORC1 activation and signaling. This is in accordance with our previously published study [15]. In a stark contrast, treatment with IGF-1 resulted in a significant increase in the phosphorylation of mTOR and p70S6K1 (Figure 6a-d). Furthermore, IGF-1 treatment completely reversed the Aβ42-induced attenuation of mTORC1 activation and signaling. To further characterize the involvement of mTORC1 in the IGF-1 induced increase in leptin expression levels, we treated the organotypic slices with rapamycin, an allosteric inhibitor of mTORC1. In the presence of rapamycin, IGF-1 was ineffective in augmenting leptin expression levels (Figure 7a-d). This suggests that mTORC1 activation and signaling are a requisite for IGF-1 induced increase in leptin expression.

### IGF-1 treatment enhances translation and increases levels of the transcription factor C-EBPα, which mediates increased leptin transcription

**Figure 8:**
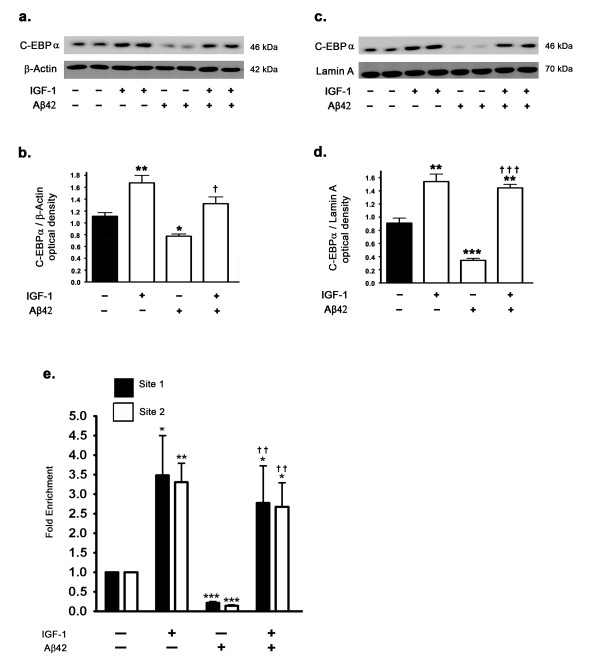
**Aβ42 reduces levels of the transcription factor C-EBPα and IGF-1 reverses the reduction in levels of C-EBPα**. (a,b) Treatment of organotypic slices with Aβ42 for 72 hours significantly decreases expression levels of the transcription factor C-EBPα in the cytosolic fraction. IGF-1 treatment increases basal levels of C-EBPα in the cytosol by ~1.6 fold and restores Aβ42-induced reduction in C-EBPα levels. (c,d) Treatment with Aβ42 for 72 hours significantly decreases levels of the C-EBPα in the nuclear fractions, suggesting that Aβ42 reduces the activation and nuclear translocation of C-EBPα. IGF-1 treatment increases the nuclear translocation of C-EBPα by ~1.8 fold and reverses the attenuation induced by Aβ42 of nuclear levels of C-EBPα. (e) ChIP analysis demonstrates that treatment with IGF-1 results in an about 3.5-fold increase in C-EBPα binding to the leptin promoter region. Aβ42 treatment results in a pronounced attenuation of C-EBPα binding to the leptin promoter region. Concomitant treatment with IGF-1 completely reverses the effects of Aβ42 on C-EBPα binding to the leptin promoter and produces a 3-fold increase in binding compared to control. *p < 0.05, **p < 0.01, and ***p < 0.001 versus control; ^† ^p < 0.05, ^† †^p < 0.01, and ^† †† ^p < 0.001 versus Aβ42.

Several lines of evidence suggest that mTORC1 regulates leptin biosynthesis at the level of translation [20-22]. In this study and our previous studies [15] we have demonstrated that treatment of organotypic slices with rapamycin, in addition to reducing leptin protein levels, also reduced leptin mRNA. This data suggests that mTORC1 may also control the translation of some of the transcription factors involved in leptin transcription. There is substantial evidence that mTORC1 translationally controls the protein levels of the transcription factor C-EBPα [34]. C-EBPα is the most abundant transcription factor regulating leptin expression in the adipose tissue [35,35,36,38]. Other transcription factors involved in leptin expression include Sp1, LP1, and AP-2β [35,36]. However, there is no general consensus suggesting regulation of these transcription factors by mTORC1 or rapamycin. A scan of the rabbit leptin gene promoter region present between 10000 nucleotides upstream and the leptin transcription initiation site using the "TFsearch" program revealed multiple C-EBPα consensus binding motifs (GenBank Accession #: NC013675). We therefore investigated the involvement of C-EBPα transcription factor in leptin expression and specifically in IGF-1-induced increase or Aβ42-induced decrease in leptin expression. Our results demonstrate that in response to IGF-1 treatment, expression and subsequent translocation of C-EBPα into the nucleus are increased as demonstrated by Western blotting (Figure 8a-d). On the other hand, treatment with Aβ42 results in a substantial attenuation of C-EBPα expression levels and subsequent translocation to the nucleus (Figure 8a-d). Remarkably, IGF-1 treatment completely reverses the attenuation induced by Aβ42 on the expression levels and subsequent nuclear translocation of C-EBPα. To correlate the nuclear levels of C-EBPα with its transcriptional activity modulating leptin expression, we next performed a ChIP assay analysis to establish the extent of binding of C-EBPα to the leptin promoter. ChIP analysis revealed a 3.5 fold increase in binding of C-EBPα in the leptin promoter region in response to IGF-1 treatment (Figure 8e). Analogous to a decrease in C-EBPα expression and subsequent nuclear translocation, Aβ42 treatment also attenuated the binding of C-EBPα to the leptin promoter. This effect induced by Aβ42 was completely reversed by concomitant IGF-1 treatment, thereby implicating C-EBPα as the molecular element utilized by Aβ42 and IGF-1 to modulate leptin expression.

**Figure 9:**
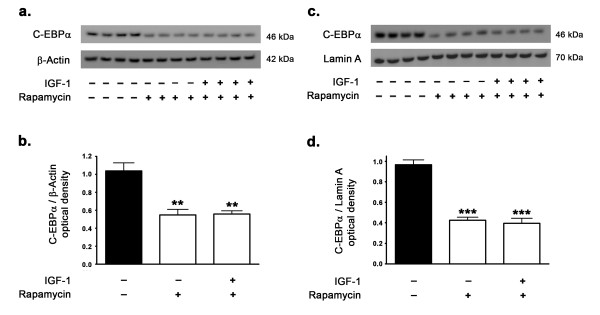
**IGF-1 fails to regulate C-EBPα expression levels in presence of the mTORC1 inhibitor rapamycin**. Western blot and densitometric analyses show that treatment of organotypic slices with the mTORC1 inhibitor rapamycin for 72 hours significantly decreases expression levels of the transcription factor C-EBPα in the cytosol (a,b) and in the nuclear fractions (c,d). IGF-1 treatment does not affect the reduction in cytosolic and nuclear levels of C-EBPα induced by rapamycin, suggesting that the upregulation of the transcription factor C-EBPα is mediated via mTORC1. **p < 0.01 and ***p < 0.001 versus control.

We also determined the extent to which mTORC1 activation and signaling is involved in the regulation of C-EBPα expression levels in the rabbit hippocampus. The mTORC1 inhibitor rapamycin significantly reduced the protein levels of C-EBPα and consequently reduced the translocation of C-EBPα into the nucleus in response to IGF-1 treatment (Figure 9a-d). Furthermore, in the presence of rapamycin, IGF-1 treatment failed to increase the expression of C-EBPα and to induce its translocation into the nucleus. This implicates C-EBPα as the mediator of the activated mTORC1-induced increase in leptin transcription. This suggests that IGF-1-induced upregulation in leptin expression is a consequence of increased binding of the transcription factor C-EBPα in the leptin promoter region and this is mediated by mTORC1 activation and signaling.

## Discussion

This study was conceived to examine the impact of Aβ on the expression of IGF-1 in the hippocampus and assess the role of leptin signaling in the modulation of IGF-1 expression. We demonstrate that Aβ42 induces a marked reduction in IGF-1 expression and treatment with the adipocytokine leptin increases the basal expression levels of IGF-1 and reverses the Aβ42-induced attenuation in IGF-1 expression levels. We further demonstrate that the inhibition of the JAK2/STAT5 underlies Aβ42 and leptin effects on IGF-1 expression, and that IGF-1 expression is mediated by the transcription factor STAT5. We also demonstrate that IGF-1 regulates leptin expression via the mTORC1 signaling pathway by a mechanism that involves the transcription factor C-EBPα. This suggests a mutual positive feedback loop between IGF-1 and leptin and indicates that both IGF-1 and leptin reinforce the expression and activation of each other.

This study demonstrates that Aβ42 inhibits the JAK2/STAT5 pathway. There is evidence that extracellular Aβ is internalized by glial cells via phagocytosis, pinocytosis, and endocytosis [39,40]. Neurons uptake Aβ from the extracellular milieu as well and this contributes to the accumulation of intraneuronal Aβ [41]. Intraneuronal accumulation of Aβ has been implicated in loss of synaptic plasticity and shown to adversely affect neuronal function and survival [42-44]. Furthermore, it has been demonstrated that intraneuronal Aβ causes memory impairment by attenuating JAK-STAT signaling in hippocampal neurons [45]. IGF-1 expression in the peripheral system is regulated by the transcription factor STAT5 [10,11,46]. The functional long-form of leptin receptor (Ob-Rb) is coupled to the JAK2/STAT5 pathway and is highly expressed in the hippocampus [47,48]. Leptin phosphorylates Ob-Rb at Tyr^1138 ^upon binding and activates the JAK/STAT signal transduction pathway [49]. Leptin binding to Ob-Rb has been shown to activate STAT5 via JAK2 [50-52]. We demonstrate in this study that Aβ42 induces a decrease in p-Tyr^1007/1008 ^JAK2 and p-Tyr^694 ^STAT5 levels, consequently reducing the nuclear translocation of STAT5 and mitigating JAK2/STAT5 signaling. On the other hand, treatment with leptin elicited a significant increase in JAK2/STAT5 activation and reversed the effects of Aβ42 on JAK2/STAT5 signaling, as shown with increased translocation of STAT5 to the nucleus. To determine the extent to which STAT5 mediates leptin effects, we treated organotypic slices with a specific inhibitor of STAT5 in the presence and absence of leptin. We found that STAT5 inhibition markedly reduced IGF-1 expression. As this attenuation of IGF-1 expression by STAT5 inhibition was not alleviated by leptin, such a result suggests that STAT5 is required for leptin-induced increase in IGF-1 expression. We further studied the IGF-1 promoter using EMSA and ChIP analyses to determine the effects of Aβ42 and leptin treatments on IGF-1 transcription and delineate the role of STAT5. We found that Aβ42 reduces the binding of STAT5 in the IGF-1 promoter region. In contrast, both EMSA and ChIP analyses showed that leptin treatment increases STAT5 binding to the IGF-1 promoter region and reverses the attenuating effects of Aβ42 on STAT5 binding in the IGF-1 promoter region. Our data strongly suggest that STAT5 plays an important role in leptin-induced increase in IGF-1 expression.

The findings that Aβ42 reduces IGF-1 expression in the brain and leptin increases the basal levels of this neurotrophic factor and reverses the Aβ-induced decrease in IGF-1 may be of relevance to AD as IGF-1 exhibits neurotrophic, neuromodulatory, neuroendocrine, and metabolic actions in the brain [53]. IGF-1 reduces amyloid burden by increasing its clearance through Aβ carrier proteins like albumin and transthyretin [1]. IGF-1 effects are transduced via the cell surface IGF-1 receptors (IGF1R) belonging to the tyrosine kinase receptor family [54,55]. The IGF1R are coupled to the PI3K/Akt/mTORC1 pathway [56]. IGF-1 signaling through IGF-1 receptors has been demonstrated to induce the activation of IRS1/PI3K/AkT/mTORC1 pathway and inhibit GSK-3β, thus attenuating tau phosphorylation in NT2N cells [57] and in primary rat cortical neurons [58]. IGF-1 precludes the β-amyloid-induced neurotoxicity in hippocampal neurons [59,60] by the activation of PI3K/Akt/mTORC1 pathway [56]. Consistent with this observation, Aβ has been shown to uncouple PI3K/Akt/mTORC1 pathway [61-63]. Furthermore Aβ42 downregulates mTORC1 signaling in SH-SY5Y neuroblastoma cells and mTORC1 signaling is attenuated in APP/PS1 mice model of AD [64].

We have demonstrated that leptin decreases both basal and Aβ42-induced increase in levels of phosphorylated tau [15]. This study shows that leptin treatment increases IGF-1 expression. We have previously shown that leptin reduces the oxysterol 27-hydroxycholesterol-induced increase in Aβ and phosphorylated tau levels [14]. Several studies have reported the pivotal role of leptin in reducing Aβ production and load [16-18] as well as tau phosphorylation [65,66]. It is thus conceivable that leptin may, in part, reduce tau phosphorylation by increasing the expression of IGF-1.

Our results demonstrating that IGF-1 regulates leptin suggest that IGF-1 and leptin mutually regulate the expression of each other. We have demonstrated previously that mTORC1 activation is necessary for leptin expression and that the mTORC1 inhibitor rapamycin inhibits leptin expression levels [15]. Furthermore, we demonstrated that Aβ42 inhibits mTORC1 activation and inhibits leptin expression [15]. It is well known that IGF-1 activates the mTORC1 signaling via the Akt signaling pathway [23,24,32]. We speculated that IGF-1 may regulate leptin expression through mTORC1 activation and may potentially reverse the deleterious effects of Aβ42 on leptin expression. To this end, we treated organotypic slices with IGF-1 in presence or absence of the mTORC1 inhibitor rapamycin. We found that IGF-1 activates mTORC1 signaling and increases leptin protein and mRNA expression levels. However, in the presence of rapamycin, IGF-1 failed to exert any effect on leptin expression, suggesting that IGF-1 regulates leptin expression via the activation of mTORC1. To determine the effects of IGF-1 treatment on Aβ42-induced downregulation of leptin expression, we incubated organotypic slices with IGF-1 and Aβ42. We found that IGF-1 alleviates the reduction induced by Aβ42 on leptin protein and mRNA expression levels.

Rapamycin is an allosteric inhibitor of mTORC1 that subsequently inhibits translation of proteins that are regulated by mTORC1, including leptin. Although, it is the consensus that rapamycin is a selective inhibitor of mTORC1, recent studies have suggested that under certain conditions, prolonged rapamycin treatment may also inhibit mTORC2 complex [67-69]. mTORC2 was identified as the kinase that activates Akt by phosphorylation at Ser^473 ^[70]. Numerous studies have demonstrated that Akt activates mTORC1 [71,72]. The fact that mTORC2 phosphorylates Akt at Ser^473^, and given that Akt activates mTORC1 signaling, indicates that mTORC2 positively regulates mTORC1 signaling. Therefore, inhibition of mTORC2 by rapamycin would result in further indirect inhibition of mTORC1, in addition to the direct allosteric inhibition of mTORC1 by rapamycin [68]. Our results showing that rapamycin also decreases the leptin mRNA levels suggest that mTORC1 is also involved in leptin transcription. To elucidate the role of mTORC1 in the regulation of leptin transcription, we determined the effects of rapamycin on the transcription factors involved in leptin expression. Evidence suggests that the transcription factor C-EBPα plays an indispensable role in leptin expression in the peripheral adipose tissue [35-38]. There are also multiple studies demonstrating the critical role of mTORC1 in the translation of C-EBPα [34]. We found that rapamycin decreases protein levels of C-EBPα in the cytosol as well as in the nucleus. We also determined the involvement of C-EBPα in the Aβ42-induced reduction and IGF-1-induced increase in leptin expression as both Aβ42 and IGF-1 regulate mTORC1 activation and signaling. Western blotting clearly showed that Aβ42 decreases C-EBPα protein levels, while IGF-1 treatment increases the basal levels of C-EBPα and reverses the Aβ42-induced reduction in C-EBPα protein levels. Additionally, ChIP analysis showed that Aβ42 treatment reduces the binding of C-EBPα to the leptin promoter, while treatment with IGF-1 induces an increase in C-EBPα to the leptin promoter.

## Conclusion

**Figure 10:**
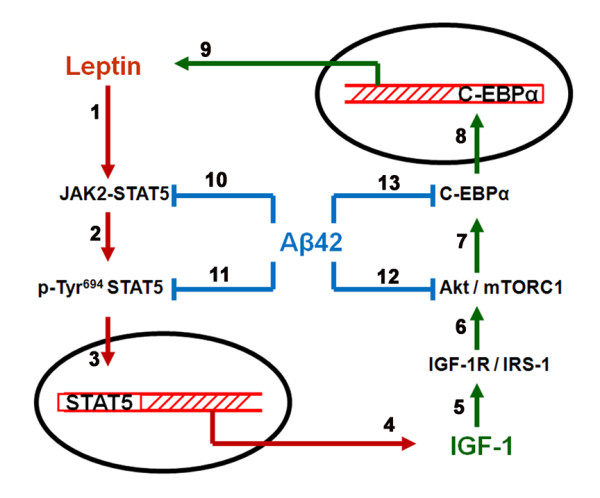
**Schematic representation of the interplay between leptin, IGF-1 and Aβ42**. Leptin activates JAK2/STAT5 pathway (1), resulting in increased levels of p-Tyr^694 ^STAT5 in the cytosol (2) and its translocation into the nucleus (3). Increased levels of p-Tyr^694 ^STAT5 in the nucleus leads to increased expression of IGF-1 (4), which through an effect on IGF-1R (5), activates the Akt/mTORC1 pathway (6). Activation of mTORC1 results in the increased expression levels of the transcription factor C-EBPα (7) and its translocation to the nucleus (8). Increased nuclear levels of C-EBPα result in its increased binding to the leptin promoter and augmentation in leptin expression (9). Aβ42 inhibits the activation of the JAK2/STAT5 pathway (10) and attenuates the levels of p-Tyr^694 ^STAT5 in the cytosol and the nucleus (11), resulting in reduced IGF-1 expression. Aβ42 also decreases mTORC1 activation and signaling (12) leading to reduction in C-EBPα levels in the cytosol and nucleus (13), effects that reduce leptin expression.

Our study is the first to demonstrate that IGF-1 and leptin mutually regulate and reinforce the expression of each other in the hippocampus, while Aβ attenuates the expression of both IGF-1 and leptin. Leptin increases the basal expression levels of IGF-1 and reverses the Aβ42-induced decrease in IGF-1 levels. Similarly, IGF-1 also increases basal expression and reverses Aβ42-induced decrease in leptin levels. The overall findings and signal transduction mechanisms involved are summarized in Figure 10. Our results are of high importance to AD studies as leptin and IGF-1 exert neuroprotective effects by reducing the accumulation of Aβ and phosphorylated tau. Understanding the cellular mechanisms involved in the regulation of leptin and IGF-1 expression levels is paramount for the search of agents that protect against AD by reducing Aβ accumulation and subsequent deleterious effects.

## Methods

### Materials

Leptin, Aβ42, and rapamycin were purchased from Sigma Aldrich (St. Louis, MO). IGF-1 peptide was purchased from Millipore (Bedford, MA). STAT5 inhibitor (573108) was obtained from Calbiochem (San Diego, CA). Hibernate A was obtained from BrainBits LLC (Springfield, IL). Membrane inserts for organotypic slices were from Millipore (Bedford, MA). The antibiotic/antimycotic agents for media (100 U/ml penicillin, and 0.05 μM/ml streptomycin) were purchased from Sigma Aldrich (St. Louis, MO). All other supplies for the culture of organotypic slices (Neurobasal medium, B27, horse serum, and glutamine) were purchased from Invitrogen (Carlsbad, CA).

### Organotypic slice preparation and treatment

We chose to use the organotypic slice system for our studies. The organotypic slice system has many advantages in that connectivity between neurons, interneurons and glia is maintained. In addition, we prepared organotypic slices from hippocampus of adult rabbits (2 year-old), a brain region and age that are relevant to the pathophysiology of AD. Additionally, rabbits have a phylogeny closer to humans than rodents [73], and their Aβ sequence, unlike that of rodents, is similar to the Aβ sequence of the human [74]. Organotypic hippocampal slices were prepared as we have previously shown [14,15] and as follows. Hippocampi from adult male rabbits (n = 6) were dissected, trimmed of excess white matter and placed into chilled dissection media composed of hibernate A containing 20% horse serum and 0.5 mM l-glutamine. Isolated tissue was placed on a wetted filter paper on the Teflon stage of a MacIlwain chopper for coronal sectioning (300 μm thick). From each rabbit hippocampi, about 50 sections were cut (100 sections per rabbit). Sections were placed in new dissection media and allowed to rest five minutes on ice before separating and plating on membrane inserts. Five sections were placed on each insert with a total of 10 inserts per hippocampus (20 inserts per rabbit). Inserts were placed in 35 mm culture dishes containing 1.1 ml growth media (Neurobasal A with 20% horse serum, 0.5 mM l-glutamine, 100 U/ml penicillin, and 0.05 μM/ml streptomycin), and warmed 30 min prior to plating to ensure complete equilibration. Slices were exposed to a humidified incubator atmosphere (4.5% CO_2 _and 35°C). Media was changed at 24 h and, at day 4, slices were switched to a defined medium consisting of Neurobasal A, 2% B27 supplement and 0.5 mM l-glutamine. At day 10, organotypic slices from each rabbit were divided into the following treatment groups: (1) vehicle, (2) 125 nM leptin, (3) 80 nM IGF-1, (4) 10 μM Aβ42, (5) 125 nM leptin + 10 μM Aβ42, (6) 80 nM IGF-1 + 10 μM Aβ42, (7) 100 nM rapamycin, (8) 100 nM rapamycin + 80 nM IGF-1, (9) 100 μM STAT5 inhibitor, and (10) 100 μM STAT5 inhibitor + 125 nM leptin. A stock solution of leptin of 62.5 μM (1 mg/ml) was prepared in sterile distilled water and diluted in media at 1:500 to a concentration of 125 nM (2 μg/ml). IGF-1 was procured as a 100 μg lyophilized powder, was dissolved in 1.11 ml sterile distilled water to yield a 12 μM (90 μg/ml) stock solution. The IGF-1 stock solution was further diluted in media at 1:150 to a concentration of 80 nM (600 ng/ml). Aβ42 peptide was dissolved in sterile distilled water to yield a 250 μM (1 mg/ml) stock solution and diluted in media at 1:25 to a final concentration of 10 μM (40 μg/ml). Rapamycin was purchased as a 2.5 mg/ml (2.74 mM) stock solution in DMSO and was diluted in media at 1:274 to yield a working stock solution of 10 μM. The rapamycin solution was further diluted at 1:100 in media to yield a final concentration of 100 nM. Each treatment was delivered into the media of 2 inserts with 5 sections from each of the 6 rabbits. Sections were harvested after 72 h of treatment. The chosen concentrations of leptin (125 nM), Aβ42 (10 μM), and rapamycin (100 nM) were based on our previously published study [15]. The concentration of leptin selected (125 nM) was based on a dose response assay conducted to determine the minimum concentration of leptin that induces phosphorylation of the leptin receptor (Ob-Rb) at Tyr^1138 ^in our organotypic slice paradigm [15]. Other studies have employed 100 nM leptin in SH-SY5Y neuroblastoma cells [16] and primary neuronal cultures [65,66]. The rapamycin concentration (100 nM) used was the empirically determined minimum concentration that inhibits mTORC1 activation in our paradigm [15]. Several other studies have utilized up to 1 μM rapamycin to inhibit mTORC1 activation and signaling in SH-SY5Y neuroblastoma cells [64,75]. The IGF-1 concentration used (80 nM) was empirically determined by a dose response assay with the concentration chosen depicting the minimum concentration that evokes IGF-1 receptor (IGF1R) phosphorylation at Tyr^1135/1136 ^residues in our organotypic slice paradigm. All animal procedures were carried out in accordance with the U.S. Public Health Service Policy on the Humane Care and Use of Laboratory Animals and were approved by the Institutional Animal Care and Use Committee at the University of North Dakota.

### Immunoprecipitation

Immunoprecipitation from tissue homogenate was performed for IGF-1 by using "Catch and Release" immunoprecipitation kit from Millipore (Bedford, MA) according to the manufacturer's protocol. Briefly, organotypic slices were homogenized in T-PER tissue protein extraction reagent (Thermo Scientific, Rockford, IL) supplemented with protease and phosphatase inhibitors. Tissue homogenate containing the equivalent to 500 μg of total protein content was incubated with 2 μg of the anti-IGF-1 goat antibody (1:500, Abcam, Cambridge, MA) overnight in the spin columns followed by elution using the denatured elution buffer containing 5% β-mercaptoethanol. 5 μL of the eluate was resolved on a SDS-PAGE gel followed by transfer onto a polyvinylidene difluoride membrane (BioRad, Hercules, CA) and incubation with IGF-1 antibody followed by development with enhanced chemiluminescence (Immun-star HRP chemiluminescent kit, Bio-Rad, Hercules, CA). Bands were visualized on a polyvinylidene difluoride membrane and analyzed by LabWorks 4.5 software on a UVP Bioimaging System (Upland, CA). Quantification of results was performed by densitometry and the results analyzed as total integrated densitometric values (arbitrary units). Rabbit liver tissue homogenate was used as a positive control, while the eluate from the column that did not contain the IGF-1 primary antibody as well as the column that was devoid of the tissue homogenate were used as the negative controls.

### Western blot analysis

Organotypic slices were homogenized in NE-PER tissue protein extraction reagent (Thermo Scientific, Rockford, IL) supplemented with protease and phosphatase inhibitors. Protein concentrations from the cytosolic and nuclear homogenates were determined with BCA protein assay. Proteins (10 μg) were separated in SDS-PAGE gels followed by transfer to a polyvinylidene difluoride membrane (BioRad, Hercules, CA) and incubation with the following monoclonal antibodies: anti-JAK2 rabbit antibody (1:1000; Cell Signaling, Boston, MA), anti-phospho (Tyr^1007/1008^) JAK2 rabbit antibody (1:200; Cell Signaling, Boston, MA), anti-STAT5 rabbit antibody (1:1000; Cell Signaling, Boston, MA), anti-phospho (Tyr^694^) STAT5 mouse antibody (1:200; Cell Signaling, Boston, MA), anti-IGF1 goat antibody (1:500; Abcam, Cambridge, MA), anti C-EBPα rabbit antibody (Active Motif, Carlsbad, CA). β-actin and lamin A were used as a gel loading control for cytosolic homogenates and nuclear homogenates respectively. The blots were developed with enhanced chemiluminescence (Immun-star HRP chemiluminescent kit, Bio-Rad, Hercules, CA). Bands were visualized on a polyvinylidene difluoride membrane and analyzed by LabWorks 4.5 software on a UVP Bioimaging System (Upland, CA). Quantification of results was performed by densitometry and the results analyzed as total integrated densitometric values (arbitrary units).

### Enzyme-linked immunosorbent assay (ELISA)

IGF-1 levels were quantified in the organotypic slices using a quantitative sandwich ELISA kit (R & D systems, Minneapolis, MN) as per the manufacturer's protocol. Organotypic slices were homogenized in T-PER tissue protein extraction reagent (Thermo Scientific, Rockford, IL) supplemented with protease and phosphatase inhibitors. Protein concentrations from tissue homogenates were determined with BCA protein assay. The tissue homogenates belonging to different treatments were further diluted in PBS to yield a protein concentration of 1 mg/ml. 20 μL of the tissue homogenate from each treatment group normalized to 1 mg/ml protein concentration was diluted 1:20 and then further 1:5 in the special buffers provided with the kit to release any IGF-1 that is bound to IGFBP's (IGF-1 binding proteins). A total of 50 μL of this 100-fold diluted homogenate was added to each well of the ELISA plate for the assay. The entire procedure for the assay was performed at 4°C. The optical density of each well was determined using a microplate reader set at 450 nm. The optical density of each well was also determined at 540 nm. The optical density values read at 540 nm were subtracted from the optical density values at 450 nm for each well to account for any optical imperfections of the ELISA plate in accordance with manufacturer's protocol. The concentrations obtained were multiplied by a factor of 100 to account for the 100-fold dilution. The IGF-1 levels were measured in triplicate for each treatment in each of the 6 rabbits. The final results are expressed as ng of IGF-1/ml of tissue homogenate.

Leptin levels were quantified in the organotypic slices using a quantitative sandwich ELISA kit (R & D systems, Minneapolis, MN) as per the manufacturer's protocol. Organotypic slices were homogenized in T-PER tissue protein extraction reagent (Thermo Scientific, Rockford, IL) supplemented with protease and phosphatase inhibitors. Protein concentrations from tissue homogenates were determined with BCA protein assay. The tissue homogenates belonging to different treatments were further diluted in PBS to yield a protein concentration of 1 mg/ml. 1 μL of the tissue homogenate from each treatment group normalized to 1 mg/ml protein concentration was further diluted 1:100 in the assay diluent buffer provided with the kit. A total of 100 μL of this diluted homogenate was added to each well of the ELISA plate for the assay. The optical density of each well was determined using a microplate reader set at 450 nm. The concentrations obtained were multiplied by a factor of 100 to account for the 100-fold dilution. The leptin levels were measured in triplicate for each treatment in each of the 6 rabbits. The final results are expressed as ng of leptin/ml of tissue homogenate.

### Quantitative Real time RT-PCR analysis

Total RNA was isolated and extracted from organotypic slices using the 5 prime "PerfectPure RNA tissue kit" (5 Prime, Inc., Gaithersburg, MD). RNA estimation was performed using "Quant-iT RNA Assay Kit" using a Qubit fluorometer according to the manufacturer's protocol (Invitrogen, Carlsbad, CA). cDNA was obtained by reverse transcribing 1 μg of extracted RNA using an iScript cDNA synthesis kit" (BioRad, Hercules, CA). The oligomeric primers (Sigma, St Louis, MO) used to amplify the leptin mRNA and IGF-1 mRNA in the hippocampal organotypic slices are enumerated in Table 1. The cDNA amplification was performed using an iQ SYBR Green Supermix kit following the manufacturer's instructions (BioRad, Hercules, CA). The amplification was performed using an iCycler iQ Multicolor Real Time PCR Detection System (BioRad, Hercules, CA). The expression of specific leptin and IGF-1 transcripts amplified were normalized to the expression of glyceraldehyde-3-phosphate dehydrogenase (GAPDH).

**Table 1:** Primers designed and used for IGF-1, leptin, IGF-1 promoter and leptin promoter

GENE	PRIMER	GenBank Accession Number	Sequence	
IGF-1	Forward	AF022961	5'-AGGCTATGGCTCCAGCATTCG-3'	RT-PCR

IGF-1	Reverse	AF022961	5'-AGTCTTGGGCATGTCAGTGTGG-3'	RT-PCR

Leptin	Forward	AF203903	5'-AGTCTTGGGCATGTCAGTGTGG-3'	RT-PCR

Leptin	Reverse	AF203903	5'-AGTCTGCCGTCCCGAAATGTG-3'	RT-PCR

IGF-1 promoter	Site-1	AC010202	5'-CCAGGGTCTCCAAGCCACTG-3'	EMSA

IGF-1 promoter	Site-2	AC010202	5'-AAATTCTAAGAAACT-3'	EMSA

IGF-1 promoter	Site-1 Forward	AC010202	5'-TTTTTCTTAGAAGTA-3'	ChIP

IGF-1 promoter	Site-1 Reverse	AC010202	5'-GATTGGTTGTGTGGCATGAG-3'	ChIP

IGF-1 promoter	Site-2 Forward	AC010202	5'-TGGCATGTTTTGAGGTTTTG-3'	ChIP

IGF-1 promoter	Site-2 Reverse	AC010202	5'-ACAAGCCCACGGGGTATGGC-3'	ChIP

Leptin promoter	Site 1 Forward	NC013675	5'-CTTCTGAGCCTTGGGCATGTCG-3'	ChIP

Leptin promoter	Site 1 Reverse	NC013675	5'-ACACACAACACCTGCCAAAA-3'	ChIP

Leptin promoter	Site 2 Forward	NC013675	5'-CACAGCACTAGGTCCAGCAG-3'	ChIP

Leptin promoter	Site 2 Reverse	NC013675	5'-ATGTGGAGTGACCCGAGAGT-3'	ChIP

### Electrophoretic Mobility Shift Assay (EMSA)

The Electrophoretic Mobility Shift Assay (EMSA) to study the STAT5-IGF-1 promoter interaction was performed using a kit from Active Motif (Carlsbad, CA) following manufacturer's protocol. Nuclear extract was prepared using NE-PER protein extraction reagent following the manufacturer's instructions (Thermo Scientific, Rockford, IL). The human IGF-1 promoter contains two STAT5 binding consensus sequences and these are evolutionary conserved across all mammalian species [30]. The rabbit IGF-1 promoter region spanning 8000 nucleotides upstream of the transcription initiation site in IGF-1 gene was scanned for STAT5 binding consensus sequences using the "TFsearch" online program that searches highly correlated sequence fragments against TFMATRIX transcription factor binding site profile database in 'TRANSFAC' databases [76,77]. The 5'-biotin labeled and unlabeled oligonucleotide probes that correspond to the STAT5 binding site in the IGF-1 promoter region (Table 1) were purchased from Sigma Aldrich (St Louis, MO). 10 μg of hippocampal nuclear proteins were incubated with either 20 femto moles of biotin labeled oligonucleotide probe or 4 pico moles of unlabelled oligonucleotide. To exhibit specificity of the oligonucleotide probes, unlabelled oligonucleotide probe was used as a specific competitor for binding reactions at a concentration of 200 fold of the concentration of the biotin labeled probe. 1 μg of Poly d(I-C) was used as a non-specific competitor for binding reactions. The resulting binding reaction mix was loaded and resolved on a 5% TBE gel (BioRad, Hercules, CA) followed by transfer onto a nylon membrane. The bands were visualized using the HRP-Streptavidin - Chemiluminescent reaction mix provided with the kit on a UVP Bioimaging System (Upland, CA).

### Chromatin Immunoprecipitation (ChIP) Analysis

ChIP analysis was performed to evaluate the extent of STAT5 and C-EBPα binding to the DNA elements in the IGF-1 promoter and leptin promoter regions respectively using "SimpleChIP^TM ^Enzymatic Chromatic IP kit" from Cell Signaling (Boston, MA). Briefly, organotypic slices from each treatment group (~100 mg) were taken and cross-linked with 1% formaldehyde for 15 min followed by the addition of 500 μL of 1.25M glycine solution to cease the cross-linking reaction. The tissue was washed with 4x volumes of 1x PBS and centrifuged at ~220g for 5 min. The pellet was resuspended and incubated for 10 min in 5 ml of tissue lysis buffer containing DTT, protease and phosphatase inhibitors. The subsequent steps to isolate the cross-linked chromatin were performed according to the manufacturer's protocol. One third of the cross-linked chromatin from each sample was set aside as "input" and the rest was subjected to immunoprecipitation. One third of the cross-linked chromatin from each sample was incubated with 5 μg of anti-phospho (Tyr^694^) STAT5 mouse antibody (Cell Signaling, Boston, MA) or with 5 μg of anti-C-EBPα mouse antibody (Cell Signaling, Boston, MA). One third of the cross-linked chromatin was also incubated with 5 μg of normal Rabbit IgG to serve as negative control. The DNA-protein complexes were collected with Protein G agarose beads and reverse cross-linked by incubation Proteinase K for 2 hours at 65°C followed by elution and purification. The relative abundance of STAT5 binding element in the STAT5 antibody precipitated chromatin and C/EBPα binding element in the C-EBPα antibody precipitated chromatin was determined by qPCR using an iQ SYBR Green Supermix kit following the manufacturer's instructions (BioRad, Hercules, CA) and sequence specific primers (Table 1). The amplification was performed using an iCycler iQ Multicolor Real Time PCR Detection System (BioRad, Hercules, CA). The fold enrichment of the STAT5 binding element and C-EBPα binding element was calculated using the ΔΔC_t _method [78] which normalizes ChIP C_t _values of each sample to the % input and background.

### Statistical analysis

The significance of differences among the samples was assessed by One Way Analysis of Variance (One Way ANOVA) followed by Tukey's post-hoc test. Statistical analysis was performed with GraphPad Prism software 4.01. Quantitative data for Western blotting analysis are presented as mean values ± S.E.M with unit value assigned to control and the magnitude of differences among the samples being expressed relative to the unit value of control. Quantitative data for ELISA analysis are presented as mean values ± S.E.M with absolute concentrations of IGF-1 and leptin reported. Quantitative data for Real time RT-PCR analysis are presented as mean values ± S.E.M, with reported values being the product of absolute value of the ratio of leptin mRNA to GAPDH mRNA multiplied by 1000000.

## Abbreviations

IGF-1: Insulin like Growth Factor-1; JAK2: Janus Kinase 2; STAT5: Signal Transducer and Activator of Transcription-5; mTORC1: mammalian Target Of Rapamycin Complex 1; mTORC2: mammalian Target Of Rapamycin Complex 2; C-EBPα: CCAAT-Enhancer Binding Protein α

## Competing interests

The authors declare that they have no competing interests.

## Authors' contributions

GM designed and performed the experiments, data analysis, and drafted the manuscript. JS and JRPP prepared the organotypic slices and samples for ChIP analysis. BD helped in experimental design and statistical analysis. OG conceived the study, designed the experiments, oversaw the entire study and wrote the final draft of the manuscript. All authors read and approved the manuscript.
